# Experiences with the implementation of a national teaching qualification in university medical centres and veterinary medicine in the Netherlands

**DOI:** 10.1007/s40037-015-0159-y

**Published:** 2015-01-22

**Authors:** Willemina M. (Ineke) Molenaar, Anneke Zanting

**Affiliations:** 1 Institute for Medical Education, University Medical Center Groningen/University of Groningen, Sector F, HPC FC 40, 9700 AD PO Box 196, Groningen, The Netherlands; 2 Centre for Education and Training, Ikazia Hospital Rotterdam, Rotterdam, The Netherlands

**Keywords:** Basic teaching qualification, University medical centres, Nationwide, Reflection on implementation

## Abstract

In 2008, a compulsory national basic teaching qualification was introduced for all university teachers in the Netherlands. At that time all eight University Medical Centres (UMCs) and the only Faculty of Veterinary Medicine had adopted or were setting up teacher development programmes. This study explores how these programmes relate to each other and to the basic teaching qualification. To gather information on teacher development programmes in the UMCs and the Veterinary Medicine Faculty an online survey was filled out by teacher development representatives from each of them. The programmes had main features in common (e.g. competency based and portfolio assessment), but differed somewhat in contents according to the local situation. Importantly, they had all been formally accepted as equivalent to the basic teaching qualification. We consider the freedom to tailor the qualifications to the medical context as well as to the local situation of the UMCs and the Veterinary Medicine Faculty one of the major success factors and the well-established collaboration between teacher development representatives of the UMCs and the Faculty of Veterinary Medicine as another. Challenges for the future include embedding the teacher development programmes in the institutional organizations and maintaining and further developing the programmes and the competencies of the qualified teachers, e.g. in a senior qualification.

## Introduction

Traditionally, no regulations about teaching competencies existed for teachers in higher education, including (veterinary) medicine [1], in sharp contrast with extensive requirements for teachers in primary and secondary education [2]. However, in the past decades teacher development programmes for university teachers were introduced and showed that these led to a significant shift from teacher-focused to student-focused teaching with improved student satisfaction scores as well as an increase in deep learning by students [3] and better performance of students [2]. In the UK a national framework for teachers in higher education was developed, providing guidelines for qualification [4]. In Norway didactic training for university teachers became mandatory in 1988 [1] and in Sweden in 2001 [5]. In the Netherlands, a national basic teaching qualification for all teachers in higher education was formalized in 2008 [6] and was incorporated in an agreement between the Ministry of Education, Culture and Science and the Association of Universities in the Netherlands in 2011. Briefly, the basic teaching qualification guarantees that the university teacher has mastered didactic competencies in the main areas of teaching (*execution of teaching—development of teaching—assessment of students—evaluation of teaching—coaching of students—organization*) and has shown sufficient capacity for further professional development. The agreement provides global criteria for content of the programme, assessment of the teachers and institutional procedures.

Medical education, at least in the Netherlands, differs from many other (undergraduate) university programmes by the widespread implementation of problem-based learning. Teachers in a problem-based or other student-centred teaching and learning environment are supposed to guide (groups of) students to construct their own knowledge rather than to transfer knowledge. The content of the problems often crosses the borders of the teachers’ own expertise and the guiding of students requires new skills. As a consequence, medical teachers are often involved in integrated, multidisciplinary teaching activities that are coordinated at a higher level and the individual teacher may not be fully and independently responsible for the full cycle of his/her teaching from conception to evaluation. Finally, the involvement of patients imposes a dual responsibility on the teacher, i.e. both for students and (future) patients. These differences may lead to frictions in the implementation of a basic teaching qualification which is developed for university teachers in general. Moreover, at the time of the introduction of the national basic teaching qualification all eight University Medical Centres (UMCs) and the only Faculty of Veterinary Medicine had already adopted or were setting up their own teacher development programmes. Representatives from all these institutions collaborated on matters related to teacher development programmes in the Special Interest Group ‘Faculty Development’ (SIG-FD) of The Netherlands Association for Medical Education [7]. The current paper explores whether the programmes of the UMCs and the Veterinary Medicine Faculty are in line with the guidelines set by the Association of Universities in the Netherlands for the basic teaching qualification [6]. In the discussion we will reflect on the implementation process and on factors which have contributed to the success of the implementation and discuss further challenges and developments.

## Material and methods

A questionnaire about teacher development programmes in the UMCs and the Veterinary Medicine Faculty was developed by three members of the SIG-FD and was adapted after discussion in the whole group. It was then completed by all nine members (100 %), thus representing all Dutch UMCs and the Veterinary Medicine Faculty programmes. The final questionnaire consisted of 103 closed questions (yes/no) covering general characteristics and the contents of the teacher development programmes, the requirements for a teacher’s portfolio and the assessment procedures. The responses were entered and analyzed in Survey Monkey (https://www.surveymonkey.com/). The date set for evaluation was 1 October 2011.

## Results

*General aspects.* All UMCs and the Veterinary Medicine Faculty have implemented a structured teacher development programme leading to a qualification which was accepted by the local university as (equivalent to) a basic teaching qualification. All programmes are competency based and include participation in didactic workshops and courses (Table 1), acquisition of teaching experience and composition of a teacher portfolio. The trainers are educational and/or content experts who often work in pairs. During the programme the trainees are coached by staff from the teacher development programme (*n* 1 = 6), by senior colleagues of their own choice (*n* = 3) or assigned to them (*n* = 1). Trainees are encouraged to collect feedback from different sources and/or to prepare audiotapes or videotapes of their teaching activities.

*Contents of the programmes*. The programmes differ somewhat in the contents of the courses (Table 1), the required teaching experiences (Table 1) and the contents of the portfolio (Table 1), largely because of local differences and/or the teaching responsibilities of the individual teachers (Table 1).

**Table 1:** Summary of the characteristics of the programmes in the UMCs

**1. Topics of didactic courses** ***(may vary between individual teachers according to their teaching tasks)*** *Large group teaching—small group teaching*—general didactic principles and theories—general principles of assessment—giving feedback—teaching philosophy of the institution—developing teaching units—use of ICT (e-learning)—assessment of knowledge—individual teaching/coaching—programme evaluation/quality control—teaching of skills—assessment of products (e.g. reports)—assessment of skills—assessment of (professional) behaviour—organization of teaching
**2. Required teaching experience** Large group teaching—small group teaching—development of teaching units—assessment of knowledge—programme evaluation—general principles of assessment—use of ICT—individual teaching/coaching—organization of teaching—bedside teaching (if applicable)
**3. Contents of portfolio** *Teaching résumé/overview of recent teaching activities*—feedback received from others with reflection—self-developed student assessments—self-developed teaching material—results of institutional evaluations—self-evaluation/analysis of strengths and weaknesses—general curriculum vitae—personal profile with personal view on teaching—feedback given to others—video registration of teaching activities—audio registration of teaching activities

The listed items represent questions that were answered positively by at least four of the UMCs and the Faculty of Veterinary Medicine (*italic:* all of them).

*Assessment.* In all programmes *assessment* is by means of a teaching portfolio (Table 1). The strictness of the prescribed structure for the portfolio was measured on a five-point scale, i.e. completely open [1] to completely prescribed [5] (range 1–4; average 2). All (*n* = 7) or part (*n* = 2) of the material in the portfolio has to be reflected on. In all programmes the portfolio is assessed by a portfolio assessment committee consisting of 2–4 members (average 2.8). Generally, for each candidate a committee is recruited from a limited pool of assessors, consisting of senior colleagues of the candidate (*n* = 5), representatives from teacher development programmes (*n* = 4), coaches of candidates not involved with the specific candidate (*n* = 3), the coach of the candidate (*n* = 1) or students (*n* = 1). Remarkably, the immediate (educational) superior of the candidates is not involved in any of the programmes. The assessment is done with the aid of a check list (*n* = 3), a scoring list (*n* = 3), a format without scores (*n* = 2) or globally (*n* = 1). A meeting with the candidate is a standard part of the procedure (*n* = 4) or not (*n* = 2) or dependent on the candidate or results of the assessment (*n* = 3). The judgment is given as satisfactory/non-satisfactory (*n* = 7), with a written report (*n* = 6) or a qualification (*n* = 1); no grades are given. Two programmes give advice for continuing development to all successful candidates, five sometimes do and two do not. Candidates whose portfolios are judged ‘unsatisfactory’ always receive specific written or oral feedback on how to adapt their portfolio in order to meet the requirements

## Discussion

Approximately 3 years after the national introduction of a basic teaching qualification for Dutch university teachers, all UMCs and the Veterinary Medicine Faculty had implemented teacher development programmes. They were found to share many features, such as a competency-based approach, the use of a teacher portfolio, a mix of training sessions and work-based experiences and portfolio-based assessment. At the same time the programmes were tailored to the profiles of the individual UMCs and Veterinary Medicine Faculty. We attribute this accomplishment largely to the longstanding collaboration and exchange of experiences of teacher development staff in the SIG-FD, which started prior to the required implementation of the basic teaching qualification. Moreover all UMC and the Veterinary Medicine Faculty qualifications are accepted as a basic teaching qualification or equivalent to that. In our view, this reflects the ‘freedom’ given by the agreement of the Association of Universities in the Netherlands to take local and disciplinary diversity into account in the implementation of a teacher qualification programme.

A number of challenges for the future can also be identified. Challenges from the point of view of the teacher development programmes themselves include ‘maintenance’ of both the programmes themselves and of the didactic competencies of formally qualified teaching staff. To assure and maintain the quality of the programmes, a national audit programme has been piloted [6]. For qualified teachers (compulsory) continuing didactic training could be introduced, either face-to-face, by distant learning or in learning communities [8, 9]. Some of the teachers with the basic teaching qualification may aspire to a career focused on teaching, including more coordinating roles. For them advanced development programmes and a senior teaching qualification have already been implemented in some institutions and will be further developed in others [6]. At the organizational level teaching in a medical setting has to compete with patient care and research, both of which are easier to quantify than teaching. In a recent paper Engbers et al. [10] analyzed the position of medical education from an organizational point of view. They suggested a framework for organizational development, in which undergraduate and postgraduate teaching has its own position as one of the core professional competencies of the organization. The challenge is to turn the professionalization of teachers from a burden to a benefit for the whole organization. In a commentary on faculty development Steinert also advocated to use faculty development as a way to initiate organizational and/or curricular changes by building consensus and generating support and enthusiasm [9].

## Conclusion

The implementation of a teaching qualification accepted as a basic teaching qualification for university teachers in all UMCs and the Veterinary Medicine Faculty has been successful. The success can at least in part be ascribed to the freedom to adapt the basic teaching qualification programme to the medical context as well as to the local situation. Challenges for the future are maintaining and further developing the programmes and the didactic competencies of teachers after their qualification and embedding the teacher development programmes in the institutional organizations.

## Footnotes

1 Numbers refer to the number of programmes to which this applies.
